# Neonatal Urine Screening Program in the Province of Quebec: Technological Upgrade from Thin Layer Chromatography to Tandem Mass Spectrometry

**DOI:** 10.3390/ijns7010018

**Published:** 2021-03-20

**Authors:** Christiane Auray-Blais, Michel Boutin, Pamela Lavoie, Bruno Maranda

**Affiliations:** Division of Medical Genetics, Department of Pediatrics, Centre de Recherche-CHUS, Faculty of Medicine and Health Sciences, Université de Sherbrooke, Sherbrooke, QC J1H 5N4, Canada; Michel.Boutin2@usherbrooke.ca (M.B.); pamela.lavoie@usherbrooke.ca (P.L.); bruno.maranda@usherbrooke.ca (B.M.)

**Keywords:** newborn screening, inborn errors of metabolism, dried urine spots, mass spectrometry, flow injection

## Abstract

The Quebec Neonatal Urine Screening Program was initiated in 1971 with overall screening inception of newborns in 1973. Forty-seven years later, over 3.5 million babies have been screened for up to 25 inborn errors of metabolism divided into two groups: (1) urea cycle disorders and organic acidurias; and (2) disorders of amino acid metabolism and transport. The main goal of this preventive genetic medicine program is the detection of treatable diseases before the onset of clinical symptoms. Urine specimens from 21-day-old babies are collected and dried on filter paper by parents at home. The participation is voluntary with a high compliance rate over the years (~90%). Specimens are analyzed by thin layer chromatography (TLC). The main objective of this evaluative research project was to assess the feasibility of a technological upgrade towards mass spectrometry. A 2.85-min flow injection method was devised, normal values established, and abnormal profiles confirmed using second-tier tests. The validated assays are sensitive, specific, and suitable for populational screening, as well as for high-risk screening laboratories. Triple H syndrome, which would not be detected in newborns by blood screening at two days of age was found to be positive in the urine of an affected patient.

## 1. Introduction

Newborn screening (NBS) for inborn errors of metabolism (IEM) was first introduced in the Province of Quebec in 1973 as part of major endeavors by the Quebec Network of Genetic Medicine (QNGM) supported by the Quebec Ministry of Health and Social Services [1]. NBS programs were initiated in Quebec City, QC, employing dried blood spots (DBS) collected at two days of age by heel prick at participating hospitals; dried urine spots (DUS) were analyzed in Sherbrooke, QC, initially collected at five days of age before discharge from the nursery [2]. Since 1981, DUS were collected at 21 days of age by parents at home, with ~90% participation over the years [3], and sent by regular mail to the screening laboratory in Sherbrooke, Quebec, where they are processed, then analyzed by thin layer chromatography (TLC). Two unidimensional migrations are typically performed on homemade TLC glass plates. A multiplex staining technique is performed where four different sprays are sequentially applied on the same plate: (1) bromocresol green for organic acids; (2) *ortho*-dianisidine for methylmalonic acid; (3) ninhydrin for amino acids; and (4) Ehrlich’s reagent for citrulline [3]. Over the years, more than 3,500,000 babies have been screened by the urinary NBS program for up to 25 inherited Mendelian disorders separated into two groups: (1) severe disorders requiring immediate therapeutic intervention (urea cycle disorders, organic acidurias) and (2) disorders of amino acid metabolism and transport, requiring surveillance and follow-up. The program has evolved tremendously since 1973. In fact, reagents and techniques were adapted and optimized over time to introduce more disorders to the NBS program, to improve detection, and achieve a better chromatographic resolution [2,3,4,5,6,7]. Moreover, different strategies were employed to maintain compliance, increase DUS quality, and reduce the rate of repeat specimens [4]. Throughout this constant evolution, the rationale for this unique-in-the-world program was oriented towards timely diagnosis and therapeutic intervention, as well as a research opportunity to improve knowledge on the natural history of selected disorders [1]. The urine NBS program complements the blood NBS program by allowing the detection of disorders that would not be identifiable in blood either because of the lack of appropriate biomarkers or the time of collection at two days of age, which might be too early to detect a biochemical abnormality. This is the case, for example, for hyperornithinemia–hyperammonemia–homocitrullinuria (Triple H) syndrome, a urea cycle disorder that is more prevalent in French-Canadians, presumably due to a founder effect. Most affected patients are homozygous for the F188del mutation in the *SLC25A15* gene [8]. Sokoro and colleagues have shown that newborns with this mutation have normal ornithine levels in blood when the DBS is collected at two days of age, leading to missed cases [9]. The NBS in blood is also hampered by the potential production of ornithine by red cell arginase during DBS drying [10], causing false positives.

The infrastructure of the NBS urine program is also a unique opportunity to pursue important research projects, always with the informed consent of parents [4]. This was the case for the five-year Quebec Neuroblastoma Screening Project (QNSP), which was initiated in 1989 [11,12,13] and funded by a grant from the National Institutes of Health (NIH). There was a growing interest for early detection of neuroblastoma patients, and some physicians in North America were advocating NBS by targeting catecholamines such as homovanillic acid (HVA) and vanillylmandelic acid (VMA) [14]. Urine screening was offered at 21 days and six months of age to all children in Quebec for a period of five years to determine if early detection could reduce mortality. Neuroblastoma screening increased the incidence of detected cases but did not decrease the incidence of unfavorable advanced-stage disease in older children and caused adverse health effects in some cases [15,16]. Conclusions drawn from the QNSP allowed authorities to make informed decisions on neuroblastoma screening worldwide. [14].

TLC has proven to be a useful technique for NBS and offers many advantages such as being simple, rapid, reproducible, and inexpensive. However, some drawbacks are also noted. The qualitative nature of the technique makes the use of external quality controls (QCs) and the participation in proficiency testing programs difficult; in addition, interferences might be encountered [17]. The different technologies used for NBS in DUS (TLC) and in DBS (tandem mass spectrometry, or MS/MS) in the Province of Quebec can also be an obstacle for an unbiased assessment to evaluate the appropriate matrix for screening specific diseases.

MS/MS, on its own or in combination with chromatography (LC-MS/MS), is now a technology of choice for NBS considering its high sensitivity and its selectivity [18,19,20,21]. After extraction from biological matrices, compounds of interest are first resolved by chromatography, then ionized and selected in the MS analyzer according to their mass-to-charge ratios. Further selectivity is obtained by the subsequent selection of a specific fragment obtained by collision-induced dissociation (CID). The use of MS/MS offers many advantages, compared to TLC, for urine NBS in Quebec: (1) possibility of absolute quantitation when calibration curves and internal standards (IS) are used; (2) direct normalization of urine concentration according to the creatinine concentration; (3) availability of external QCs and proficiency testing for the majority of the targeted biomarkers; (4) greater flexibility for the addition of disorders to be screened; and (5) better selectivity, thus less interferences. The objective of this research project was thus to evaluate the feasibility of proceeding to a technological upgrade from TLC to MS/MS for the urine NBS program in the Province of Quebec. The targeted biomarkers and associated IEM, which are part of this technological upgrade, are shown in Table 1. In order to achieve this objective, the following stepwise tasks were undertaken: (1) Devise a high-throughput method, referred to as the screening test, allowing the analysis of 500 urine specimens collected on filter paper per day; (2) devise and/or validate second-tier tests for amino acids, and relevant organic acids, acylglycines, pyrimidines, and biomarkers of creatine synthesis and transport disorders [22], aiming to confirm the abnormal results obtained at the screening test; (3) establish normal reference values for each biomarker analyzed; (4) perform the comparison of the two second-tier test methodologies with gold standard ion-exchange chromatography method for amino acids and gas-chromatography for organic acids; and (5) perform the analyses of known IEM cases compared to controls. Two second-tier tests were devised and/or validated: (1) A complete profile of amino acids (45 molecules) using the Kairos Amino Acid kit (Waters Corp.), referred as the “Kairos amino acid” second-tier test, was implemented and validated [23]; and (2) an in-house method for the multiplex analysis of all the other targeted biomarkers, referred as the “organic acids” second-tier test, was developed, and validated.

## 2. Materials and Methods

### 2.1. Ethics Approval

This project was approved by the Research Ethics Board (REB) at the Faculty of Medicine and Health Sciences at the Centre intégré universitaire de santé et de services sociaux de l’Estrie-Centre hospitalier universitaire de Sherbrooke (CIUSSSE-CHUS) (Project #2019-3173, date of the approval: 1 April 2019).

### 2.2. Overview of Current and Future Sample Processing and Interventions

**Figure 1:**
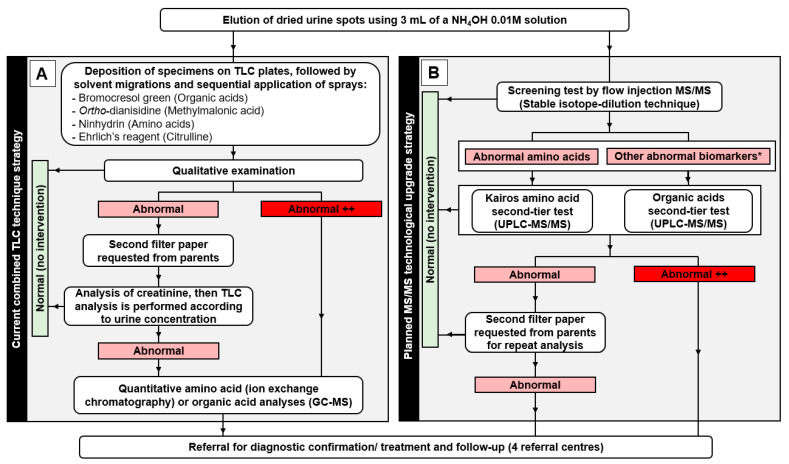
Flowsheet diagram describing (**A**) the current steps from sample processing to thin layer chromatography (TLC) analyses and related interventions for the newborn screening (NBS) program and (**B**) the expected steps from sample processing to tandem mass spectrometry analyses as part of the planned technological transfer of the NBS program. * Other abnormal biomarkers: creatine, creatinine, guanidineacetic acid, 3-methylcrotonylglycine, orotic acid, methylmalonic acid, glutaric acid, 3-hydroxyisovaleric acid, malonic acid, 3-hydroxyglutaric acid, isovalerylglycine, 2-methylcitric acid, uracil, propionylglycine, 3-hydroxypropionic acid, and homogentisic acid.

A flowsheet diagram of the NBS program in its actual form and future changes for mass spectrometry analysis is depicted in Figure 1. The collection of urine specimens on filter paper and the elution process will remain the same.

### 2.3. Urine Specimens Collected on Filter Paper

Specimens are collected at 21 days of age by parents as previously described [3]. Briefly, a urine collection kit containing a Whatman-GE 903 filter paper, absorbent pads, an identification form, and the instructions for the urine collection procedure is provided to parents before their departure from the hospital, along with a leaflet containing relevant information on the program. The absorbent pad is deposited in the baby’s diaper. When the absorbent pad is completely soaked with urine, the filter paper is pressed against it until saturation and left to dry on a clean counter. The dried urine specimen is then sent by regular mail in a pre-addressed envelope to the laboratory for analysis.

Anonymized urine specimens collected as part of the urine NBS program were analyzed to establish the normal reference values of all biomarkers under study at 21 days of age. Urine specimens from positive cases at 21 days of age were also analyzed when available: combined malonic and methylmalonic aciduria (CMAMMA) (*n* = 2), glutaric aciduria type 1 (*n* = 2), Triple H syndrome (*n* = 1), 3-methylcrotonylglycinuria (*n* = 2), methylmalonic aciduria (*n* = 4), hyperargininuria (*n* = 2), argininosuccinic aciduria (ASA) (*n* = 1), citrullinuria (*n* = 2), and cystinuria (*n* = 4). Positive cases were confirmed by a physician based on clinical manifestations, and molecular and biochemical analyses.

Urine specimens from positive cases which were not detected as part of the actual NBS program (older than 21 days of age) were also analyzed to validate the methods: Creatine transporter deficiency (*n* = 1), guanidineacetate methyltransferase deficiency (GAMT) (*n* = 1), alkaptonuria (*n* = 1), isovaleric aciduria (*n* = 1), propionic aciduria (*n* = 1), and homocystinuria (*n* = 1).

### 2.4. Reagents

Creatinine anhydrous (≥98.0%), creatine anhydrous (≥98.0%), guanidineacetic acid (99%), L-ornithine monohydrochloride (99%), L-citrulline (98%), L-arginine (98%), L-cystine (98.5–101%), L-homocystine (98%), orotic acid anhydrous (98%), methylmalonic acid (99%), glutaric acid (99%), malonic acid (99%), N-isovalerylglycine (≥98.0%), uracil (99%), N-propionylglycine (≥98.0%), argininosuccinic acid disodium salt hydrate (≥80.0%), and L-lysine (98%) were from Sigma-Aldrich Inc. (Saint-Louis, MO, USA). Homogentisic acid, 3-methylcrotonylglycine, 3-hydroxyisovaleric acid (97%), 3-hydroxyglutaric acid (98%), 3-hydroxypropionic acid sodium salt (98%), homogentisic acid−^13^C_6_, L-arginine−^13^C_6_−^15^N_4_ (98.0%), 3-hydroxyisovaleric acid-D_8_, and 3-hydroxypropionic acid sodium salt-D_4_ were from Toronto Research Chemicals Inc. (Toronto, ON, Canada). Creatinine-D_3_ (99 atom % D), creatine-D_3_ monohydrate (99 atom % D), guanidineacetic acid-D_2_ (97 atom % D), ornithine-D_7_ (HCl) (98 atom % D), citrulline-D_7_ (98 atom % D), N-(3-methylcrotonylglycine)-D_2_ (98 atom % D), cystine-D_6_ (98 atom % D), homocystine-D_8_ (98 atom % D), methylmalonic acid-D_3_ (99 atom % D), glutaric acid-D_4_ (98 atom % D), 3-hydroxyglutaric acid-D_5_ (98 atom % D), N-isovalerylglycine-D_9_ (98 atom % D), N-propionylglycine-2,2-D_2_ (98 atom % D), lysine-D_3_ (98 atom % D), 2-Methylcitric acid (98.5%), and 2-methylcitric acid-D_3_ (99 atom % D) were from C/D/N Isotopes Inc. (Pointe-Claire, QC, Canada). Orotic acid−^15^N_2_ (98.0%), malonic acid-1,3−^13^C_2_ (99%), and uracil−^15^N_2_ (98.0%) were from Cambridge Isotope Laboratories Inc. (Andover, MA, USA). The Kairos Amino Acid kits were provided by Waters Corporation (Milford, MA, USA). Formic acid (FA) was from Acros Organics (Morris Plain, NJ, USA). Optima LC/MS grade water and ACS reagent grade ammonium formate were from Fisher Scientific (Fair Lawn, NJ, USA). HPLC grade methanol (MeOH) and acetonitrile (ACN) were from EMD Chemicals Inc. (Damstadt, Germany). A.C.S. grade ammonium hydroxide (28%) was from ACP Chemicals (Montreal, QC, Canada). Synthetic human urine was from BioIVT (Hicksville, NY, USA).

### 2.5. Preparation of Solutions

#### 2.5.1. Standard Stock Solutions

Standard stock solutions were prepared in water, and concentrations are shown in Table S1. Precautions were taken to ensure that the dissolution was complete for L-cystine and L-homocystine. The L-cystine solution was agitated for two hours at 40 °C, then overnight at room temperature, while the L-homocystine solution was agitated overnight at room temperature only. These stock solutions were used in the screening test to calibrate the concentration of the isotopically labeled standards. All these stock solutions, except for ornithine, citrulline, arginine, cystine, homocystine, and lysine, were also used for the organic acids second-tier test to prepare standard working solutions. They were stable for at least 18 months when stored at −20 °C.

#### 2.5.2. Standard Working Solutions

##### Kairos Amino Acid Second-Tier Test

Six vials of lyophilized standards were provided as part of the Kairos Amino Acid kit [23]. These standards were resuspended in 0.5 mL of 0.1 M hydrochloric acid (HCl) and mixed at room temperature for 30 min on an orbital shaker (150 RPM). The analyte concentrations in these standard working solutions are available in Table S2 and were stable at −30 °C for at least 30 days.

##### Organic Acids Second-Tier Test

Calibrator 6 working solution was prepared in water using the stock solutions described in Table S1. Calibrator 6 was then serially diluted in water according to Table S3 (dilution factors = 1:1; 1:1; 1:2,5; 1:5; 1:4) to obtain calibrators 5 to 1. These working solutions were stable at −30 °C for at least three months.

#### 2.5.3. Internal Standard Solutions

##### Screening Test

The mixture of IS used for the analysis of urine samples and QCs was prepared in water according to Table S4. The stock solution concentrations of the isotopically labeled standards were measured using the stock solutions of the light versions of these molecules (Table S1) to compensate for the effect of the isotopes on the fragmentation pattern, for the isotopic impurities, and for the error caused by weighing small amounts of compounds. The concentrations of the different IS in the mixture were chosen to have less than 2% interferences from the urine samples according to the analysis of 96 urine samples from healthy newborns without IS.

##### Kairos Amino Acid Second-Tier Test

A mixture of lyophilized IS was provided as part of the Kairos Amino Acid kit [23]. After resuspension with 2 mL of water, the solution was mixed at room temperature for 10 min on an orbital shaker (150 RPM). The volume was then completed to 10 mL with 10% sulfosalicylic acid. This working solution was stable at −20 °C for at least 30 days.

##### Organic Acids Second-Tier Test

The IS working solution used as part of the Organic acids second-tier test was prepared in water according to Table S5 and showed no sign of degradation when kept at −20 °C for at least three months.

#### 2.5.4. Quality Controls

Four internal quality assurance (IQA) QCs were purchased from the European Research Network for Evaluation and Improvement of Screening, Diagnosis, and Treatment of Inherited Disorders of Metabolism (ERNDIM): (1) control organic acids in urine; (2) control purines and pyrimidines in urine; (3) control special assays in urine (levels 1 and 2); and (4) control amino acids in serum (Levels 1 and 2). The control Amino Acids in serum (Levels 1 and 2) were used to evaluate the Kairos amino acid second-tier test only, considering that this assay includes a protein precipitation step and is suitable for both urine and plasma analyses. It is noteworthy to mention that there is no control material available from ERNDIM for the analysis of amino acids in urine. In-house QCs were also prepared for the screening test and second-tier tests and the preparation is described in the following sections.

##### Screening Test

Two in-house QCs containing creatinine (100 µM) and the 22 other biomarkers at concentrations corresponding approximately to one time (QC1X) and five times (QC5X) the normal cut-off values were prepared in water according to Table S6.

##### Kairos Amino Acid Second-Tier Test

Lyophilized QCs were provided as part of the Kairos Amino Acid kit at two levels of concentration (low range and high range concentrations). Lyophilized QCs were resuspended in 0.5 mL of 0.1 M HCl and mixed at room temperature for 30 min on an orbital shaker (150 RPM). Concentrations are shown in Table S2. QC working solutions were stable at −20 °C for at least 30 days.

##### Organic Acids Second-Tier Test

Four in-house QC working solutions (LOQ: limit of quantitation; LQC: low range quality control (3X LOQ); MQC: mid range quality control (80X LOQ); HQC: high range quality control (160X LOQ)) containing creatinine, and the 16 other biomarkers were prepared in water and concentrations as shown in Table S3.

### 2.6. Sample Preparation

Upon reception, urine specimens dried on filter paper are examined carefully under ultraviolet light to verify if there is enough urine for analysis and for the presence of contaminants such as feces. A 5-cm diameter disk of urine dried on filter paper is then punched from an area free of contaminants, folded in half, deposited in a 20-mL glass bottle, and eluted with 3 mL of a NH_4_OH 0.01 M solution using a Gyratory Shaker (Model G2, New Brunswick Scientific Co., Edison, NJ, USA) for 10 min at 300 RPM [3]. This eluate is then analyzed by the screening test, and eventually by one or both second-tier tests, when abnormal results are obtained.

#### 2.6.1. Screening Test

Twenty-five microliters of urine sample or QC are mixed with 250 µL of the IS working solution in a 1 mL well from a 96-well filtration plate (AcropreoAdv 0.2 µm WWPTFE, Pall Corporation, Port Washington, NY, USA). Thereafter, the samples are filtrated using a centrifuge (Beckman Coulter, Model, J-E, Brea, CA, USA) at 829× *g* for 2 min, and retrieved in a 1 mL 96-well collection plate (Waters Corp.).

#### 2.6.2. Kairos Amino Acid Second-Tier Test

Specimens were prepared according to the procedure provided with the Kairos Amino Acid kit [23].

For the protein precipitation procedure, 50 µL of standard working solution, QC working solution, or eluted urine sample was added to a 1.5 mL microcentrifuge tube. Fifty microliters of the IS working solution (containing sulfosalicylic acid to precipitate proteins) was added, then the solution was homogenized using a vortex mixer for five seconds. Then, fifty microliters of water (for calibrators and patient samples) or matrix (for QC samples) was added, and the solution was homogenized using a vortex mixer for five seconds before centrifugation for 15 min at 9400 g. For the derivatization procedure, 10 µL of supernatant was added to a Max Recovery Vial (Waters Corp.) containing 70 µL of borate buffer. After the addition of 20 µL of the AccQ▪Tag reagent (Waters Corp.), the solution was homogenized using a vortex mixer for five seconds. After one minute at room temperature, the vials were heated for 10 min at 55 °C in a gas chromatography oven, and 2 µL was injected in the ultra-high performance liquid chromatography tandem mass spectrometry (UHPLC–MS/MS) system.

The calibrators were prepared without matrix, considering that endogenous levels of the analytes are found in normal urine specimens and in synthetic human urine for some molecules. Nevertheless, matrix effects were evaluated accordingly. A pool of urine specimens from babies at 21 days of age, collected on filter paper then eluted with 3 mL of a NH_4_OH 0.01 M solution, was used as the matrix for the QC samples.

#### 2.6.3. Organic Acids Second-Tier Test

Specimens were prepared by stable isotope-dilution. In a 2-mL vial containing an insert, 200 µL of the IS working solution was added, along with 25 µL of standard working solution, QC working solution, or eluted urine sample. Then, 25 µL of H_2_O (for calibrators and patient samples) or matrix (QC samples) was added, and the solution was homogenized using a vortex mixer for five seconds. Ten microliters were injected in the UHPLC-MS/MS system.

The calibrators were prepared without matrix, considering that endogenous levels of the analytes are found in normal urine specimens and in synthetic human urine for some molecules. Nevertheless, matrix effects were evaluated accordingly. Regarding the matrix used in the QC samples, synthetic human urine was diluted (1:25) to mimic urine from a 21-day old baby, then 1 mL was added to a 5-cm diameter filter paper disk, dried, and eluted with 3 mL of a NH_4_OH 0.01 M solution.

### 2.7. Instrumentation and Parameters

#### 2.7.1. Screening Test

The samples were analyzed using an Acquity I-Class UHPLC system equipped with a sample organizer for management of ten 1 mL 96-well plates (Waters Corp.). The system works with a “Flow through needle injector” operated in the flow injection mode. A 0.2 µm prefilter connected to a stainless-steel union was used instead of a chromatographic column. The UHPLC system was coupled to a Xevo TQ-S Micro mass spectrometer (Waters Corp.). The UHPLC and MS parameters are shown in Table 2 and the multiple reaction monitoring (MRM) transitions in Table S7.

Uracil was analyzed in positive electrospray due to the interference of an in-source fragment of orotic acid when analyzed in negative electrospray. The 3-hydroxypropionic acid fragment was chosen to prevent the interference of lactic acid. The most sensitive fragment of 2-methylcitric was not chosen due to the presence of an unidentified interference. As per our experience, the methylmalonic acid fragment and collision energy were optimized to minimize the interference of succinic acid [24]. With the optimized parameters, the response factor of methylmalonic acid is 34 times the one of succinic acid. The most sensitive fragment of homogentisic acid was not chosen due to an unidentified molecule interfering with its IS. The developed MRM method does not allow the distinction between 3-hydroxyglutaric acid and 2-hydroxyglutaric acid.

#### 2.7.2. Kairos Amino Acid Second-Tier Test

The Kairos amino acid second-tier test was performed using a separate Acquity I-Class Xevo TQ-S Micro UHPLC-MS/MS system. Amino acids were analyzed according to the procedure provided with the Kairos Amino Acid kit [23]. This kit allows the confirmation of elevated levels of the amino acids detected as part of the screening test (arginine, argininosuccinic acid, citrulline, ornithine, cystine, lysine, and homocystine), but also provides a complete amino acid profile including 45 analytes. Electrospray ionization was used in positive ion mode. The chromatography column was a CORTECS UPLC C_18_ column of 2.1 mm× 150 mm and 1.6 μm particle size (Waters Corp.) with an on-line pre-filter (0.2 μm). The UHPLC and MS parameters are presented in Table S8 and the MRM functions in Table S9. Lysine^13^C_6_,^15^N_2_ was used as the IS of hydroxyproline instead of Arginine^13^C_6_,^15^N_4_ to achieve a better correction.

#### 2.7.3. Organic Acids Second-Tier Test

The organic acids second-tier test was performed on the same system as for the Kairos amino acid second-tier test. Electrospray ionization was used in both positive and negative ion modes. The chromatography column was an Atlantis PREMIER BEH C_18_ AX VanGuard FIT of 2.1 mm × 100 mm and 1.7 μm particle size. The UHPLC and MS parameters are presented in Table 3 and the MRM functions in Table S10. Spare functions were programmed to add new biomarkers in the future without jeopardizing the dwell times established during the validation. The M + 1 peak was used for the detection of creatinine, creatine, and 3-methylcrotonylglycine, to avoid signal saturation.

### 2.8. Data Analysis

#### 2.8.1. Screening Test

The mass spectrometry results were analyzed using the NeoLynx software integrated to MassLynx v4.2 (Waters Corp.). NeoLynx was used to calculate the biomarker concentrations using the signal measured for the biomarkers and their IS, and the concentration of the IS contained in the samples. NeoLynx was programmed to measure the signal of the different ions analyzed from scan 16 to scan 77. For creatinine, the confirmation signal ratio obtained using two different product ions was used to detect the presence of an unknown endogenous interference significantly affecting the creatinine concentration measured in approximately 0.38% of the samples. The creatinine concentration measured in each sample was entered in the supplementary column (S1) of the MassLynx sample list to allow the normalization of all the biomarkers with creatinine in NeoLynx even if they were not analyzed in the same acquisition function.

Since the IS for argininosuccinic acid was not available at an affordable cost for screening, the cystine IS was used for this molecule because, among all the IS used, it was the one with the most similar ionization behavior profile compared to argininosuccinic acid. To evaluate the argininosuccinic acid/cystine relative response factor (RRF), a solution containing 0.1 mM of both molecules was injected, and the signal obtained for argininosuccinic acid was divided by the signal obtained for cystine-D_6_. The NeoLynx results were obtained using the following formulas:

1. A = (m_creat_/m_(IS-creat)_)*IS_creat_
2. B = (1000*(m_biomarker_/m_(IS-biomarker)_)*IS_biomarker_)/A
3. C = (1000*(m_arginino_/m_(IS-cystine)_)*IS_cystine_/RRF_(arginino/cystine)_)/A
4. D = ((m_(creat-confirmation)_/m_(IS-creat-confirmation_)*IS_creat_)/A
5. E = ((m_guanidine_/m_(IS-guanidine)_)*IS_guanidine_)/((m_creatine_/m_(IS-creatine)_)*IS_creatine_)

- A = Creatinine concentration (µM)
- m_creat_ = Signal of creatinine
- m_(IS-creat)_ = Signal of creatinine internal standard
- IS_creat_ = Creatinine internal standard concentration (µM)
- B = Biomarker concentration normalized with creatinine (µmol/mmol creatinine)
- m_biomarker_ = Signal of the biomarker analyzed
- m_(IS-biomarker)_ = Signal of the internal standard of the biomarker
- C = Argininosuccinic acid concentration normalized with creatinine (µmol/mmol creatinine)
- m_arginino_ = Signal of argininosuccinic acid
- m_(IS-cystine)_ = Signal of the cystine internal standard
- IS_cystine_ = Cystine internal standard concentration (µM)
- RRF_(arginino/cystine)_ = Relative response factor (argininosuccinic acid/cystine)
- D = Creatinine confirmation ratio (no unit)
- m_(creat-confirmation)_ = Signal of creatinine confirmation ion
- m_(IS-creat-confirmation)_ = Signal of creatinine internal standard confirmation ion
- E = Ratio guanidineacetic acid/creatine (no unit)
- m_guanidine_ = Signal guanidineacetic acid
- m_(IS-guanidine)_ = Signal guanidineacetic acid internal standard
- IS_guanidine_ = Guanidineacetic acid internal standard concentration (µM)
- m_creatine_ = Signal of creatine
- m_(IS-creatine)_ = Signal of creatine internal standard
- IS_creatine_ = Creatine internal standard concentration (µM)

Flags were programmed in NeoLynx to point out samples considered as abnormal and necessitating second-tier test analyses. No background subtraction was performed.

#### 2.8.2. Kairos Amino Acid Second-Tier Test

Quantitation was achieved using six-point calibration curves. A linear curve type was chosen for cystine, homocystine, lysine, and arginine, while the best results were obtained using a second order curve type for citrulline, argininosuccinic acid, and ornithine. A 1/X weighting factor was applied, and the origin was excluded for all analytes. Quantitation was performed using the response factor, and the IS used for each molecule are shown in Table S9. Data processing was achieved using TargetLynx Application Manager, an option with MassLynx (Version 4.2) software (Waters Corp.). Variations due to the quantity of urine available on filter paper and its concentration were minimized by normalizing biomarker results as ratios to creatinine. Creatinine values obtained as part of the screening test were used when the confirmation signal ratio was acceptable (between 1.15 and 1.51). Otherwise, creatinine measurement was performed as part of the Organic acids second-tier test.

#### 2.8.3. Organic Acids Second-Tier Test

Quantitation was achieved using six-point calibration curves. Best results were obtained using a second order curve type for all molecules. A 1/X weighting factor was applied, and the origin was excluded for all analytes. Quantitation was performed using the response factor, and each molecule had its corresponding heavy-labeled IS. Data processing was achieved using TargetLynx Application Manager, an option with MassLynx (Version 4.2) software (Waters Corp.). Variations due to the quantity of urine available on filter paper and its concentration were minimized by normalizing biomarker results as ratios to creatinine.

### 2.9. Validation

#### 2.9.1. Screening Test

Intraday (*n* = 5) and interday (*n* = 5) precision and accuracy assays were evaluated using the ERNDIM IQA QCs in urine and the in-house QCs (QC1X and QC5X, Table S6). The limits of detection (LOD) were not evaluated, since the normal reference values were significantly over the limits of detection. A total of 8227 urine samples from newborn controls were analyzed to establish normal reference values for all the biomarkers analyzed.

#### 2.9.2. Kairos Amino Acid Second-Tier Test

The Kairos amino acid kit was originally devised and validated on an Acquity UHPLC I-Class/Xevo TQ-S Micro system [23], which is the same system used in our laboratory. Nevertheless, a partial validation was performed following the implementation of the assay in our laboratory. Intraday (*n* = 5 replicates) and interday (*n* = 5 days × 3 replicates each day) precision and accuracy assays were evaluated using fortified QCs at two levels of concentration in eluted urine specimens from 21-day old babies (LQC, and HQC). An unfortified QC (QC0) was also prepared in the same eluted urine specimen to correct for endogenous concentrations. Accuracy was evaluated by comparing measured concentrations back calculated from the calibration curves, and corrected for endogenous levels, to the theoretical concentration, and expressed as %bias. Precision was evaluated by measuring the percentage of relative standard deviation (%RSD) on replicates. Accuracy was also evaluated using the material Control Amino Acids in serum (Levels 1 and 2), but the following compounds were not part of this control material: sarcosine, alpha aminoadipic acid, homocitrulline, allo-isoleucine, argininosuccinic acid, beta alanine, beta aminobutyric acid, homocystine, gamma aminobutyric acid, methylhistidine, carnosine, ethanolamine, phosphoethanolamine, hydroxylysine, glycyl proline, s-sulfocysteine, anserine, kynurenine, and tryptophan. The six-point calibration curve was evaluated for each molecule according to the coefficient of determination (R^2^), and examination of residuals. LODs and LOQs were defined as three- and ten-times the analyte response SD at low concentration (*n* = 8), respectively, divided by the slope of their respective calibration curve. Matrix effects were assessed in QCs prepared in six different matrices by evaluating precision and accuracy. Carry-over was verified following the injection of a blank sample after the most concentrated calibrator of the calibration curve. Reference ranges (5th–95th percentiles) were established for babies at 21 days of age following the analysis of 50 urine specimens collected on filter paper.

#### 2.9.3. Organic Acids Second-Tier Test

The organic acids second-tier test was devised in-house and validated according to the latest Bioanalytical Method Validation Guidance for Industry document from the U.S. Food and Drug Administration (FDA) [25].

Three accuracy and precision (A&P) runs (*n* = 5 replicates) were performed over three days to evaluate precision and accuracy using eluted synthetic human urine fortified at four levels of concentration (LOQ, LQC, MQC, HQC). Endogenous concentrations were subtracted following the analysis of the unfortified eluted synthetic human urine specimen (QC0). Other validation runs (*n* = 5) were performed at three levels of concentration (LQC, MQC, HQC) in duplicates. For the A&P runs, accuracy was evaluated by comparing measured concentrations back calculated from the calibration curves and corrected for endogenous levels to the nominal concentration and expressed as %bias. Precision was evaluated by measuring %RSD on replicates. For the other validation runs, the measured values were compared against the nominal values and the %bias was calculated. For creatine, creatinine, and 3-hydroxypropionic acid, the endogenous value in synthetic human urine was higher than 50% of the nominal values of LOQ and LQC for these molecules. QCs at LOQ and LQCs were thus prepared in water instead of synthetic human urine for these molecules.

The six-point calibration curve was evaluated for each molecule according to the coefficient of determination (R^2^) and examination of residuals. The first calibrator of the calibration curve is the LOQ. LOQs were defined to obtain a signal-to-noise ratio ≥10. The LOD was defined as three-times the analyte response SD at low concentration (*n* = 5), divided by the slope of their respective calibration curve. Matrix effects were assessed in QCs (mid-range concentration) prepared in six different eluted 21-day old baby urine samples by evaluating precision and accuracy, always correcting for endogenous values. Carry-over was verified following the injection of a blank sample after the most concentrated calibrator of the calibration curve. The selectivity was evaluated by ensuring that the interferences in control urine specimens did not exceed 5% of the average IS signal in the calibrators and QCs. The specificity was verified for known isobaric interferences, which were described in the literature [26]: lactic acid for 3-hydroxypropionic acid, succinic acid for methylmalonic acid, ethylmalonic acid for glutaric acid, 2-hydroxyglutaric acid for 3-hydroxyglutaric acid, tiglylglycine for 3-methylcrotonylglycine, and methylbutyrylglycine for isovalerylglycine. Reference ranges (5th–95th percentiles) were established in babies at 21 days of age following the analysis of 50 urine specimens collected on filter paper. The external control material used to evaluate accuracy were: (1) control organic acids in urine; (2) control purines and pyrimidines in urine; and (3) control special assays in urine (Levels 1 and 2). Moreover, considering that stability experiments were not done before, the stability of extracted urine specimens collected on filter paper was evaluated at room temperature (22 °C) for one week, in a refrigerator (4 °C) for one week, in a freezer (−30 °C) for nine weeks, at −80 °C for nine weeks, and after three freeze–thaw cycles. The stability was also evaluated in processed specimens left in the UHPLC autosampler for one week. The recovery of the analytes following their elution from the filter papers was evaluated. Finally, the feasibility to perform a 1:20 dilution (20 µL of sample + 380 µL of water) if a urine specimen had biomarker concentrations outside the range of the calibration curve was evaluated (*n* = 5 replicates).

## 3. Results

### 3.1. Chromatograms

#### 3.1.1. Screening Test

**Figure 2:**
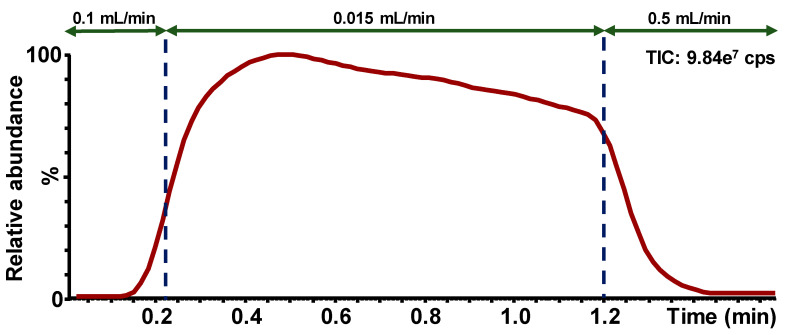
Example of ion chromatogram obtained for the screening test using the flow injection mode; Function 1 (ESI+); Flow rate modifications during the sample run are presented by the arrows; Cps = counts per second.

Figure 2 shows an example of ion chromatogram (Function 1, ESI+) obtained from the screening test using the flow injection mode. The analysis time, including the injection process, is 2.85 min.

#### 3.1.2. Kairos Amino Acids Second-Tier Test

**Figure 3:**
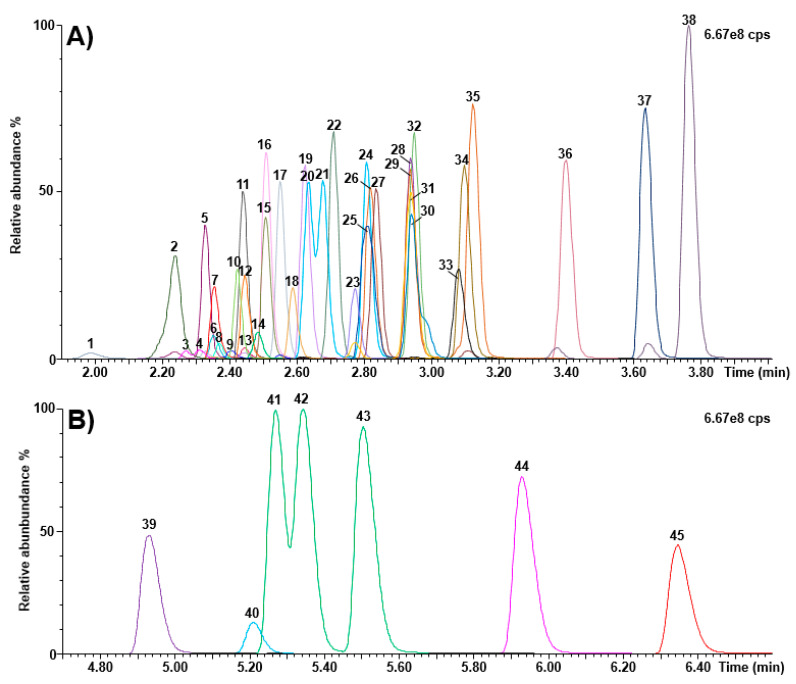
Chromatograms (calibrator 5) obtained as part of the Kairos amino acid second-tier test. (**A**) retention time window ranging from 1.90 to 4.00 min; (**B**) retention time window ranging from 4.70 to 6.60 min. 1: histidine; 2: 4-hydroxyproline; 3: 3-methylhistidine; 4: 1-methylhistidine; 5: asparagine; 6: arginine; 7: phosphoethanolamine; 8: carnosine; 9: anserine; 10: taurine; 11: serine; 12: glutamine; 13: argininosuccinic acid; 14: s-sulfocysteine; 15: glycine; 16: ethanolamine; 17: aspartic acid; 18: citrulline; 19: glutamic acid; 20: sarcosine; 21: beta-alanine; 22: threonine; 23: homocitrulline; 24: alanine; 25: hydroxylysine; 26: gamma-aminobutyric acid; 27: alpha aminoadipic acid; 28: L-ornithine; 29: beta-aminobutyric acid; 30: cystathionine; 31: glycyl proline; 32: proline; 33: cystine; 34: lysine; 35: alpha-aminobutyric acid; 36: tyrosine; 37: methionine; 38: valine; 39: homocystine; 40: kynurenine; 41: isoleucine; 42: allo-isoleucine; 43: leucine; 44: phenylalanine; 45: tryptophan; cps: counts per second.

Figure 3 shows chromatograms obtained following the injection of calibrator 5. The method run-time and the total analysis time between injections were 9 and 10 min, respectively.

#### 3.1.3. Organic Acids Second-Tier Test

**Figure 4:**
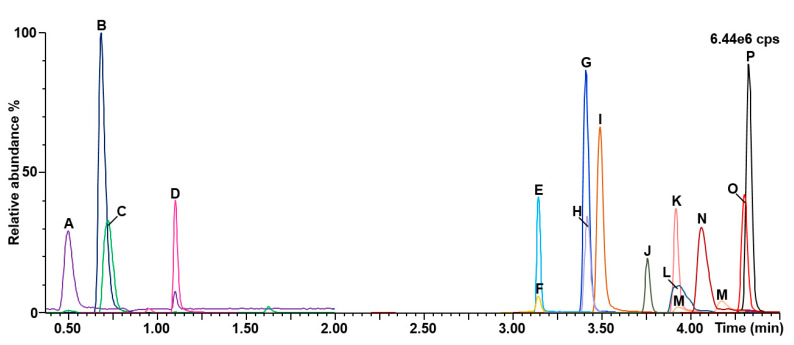
Chromatograms obtained for the organic acids second-tier test. A: Creatinine; B: Guanidineacetic acid; C: Creatine; D: Uracil; E: 3-hydroxypropionic acid; F: 3-hydroxyglutaric acid; G: glutaric acid; H: propionylglycine; I: 3-hydroxyisovaleric acid; J: homogentisic acid; K: orotic acid; L: malonic acid; M: 2-methylcitric acid; N: methylmalonic acid; O: 3-methylcrotonylglycine; P: isovalerylglycine; cps: counts per second.

A representative chromatogram is shown in Figure 4. The method run-time and the total analysis time between injections were eight and nine minutes, respectively.

**Figure 5:**
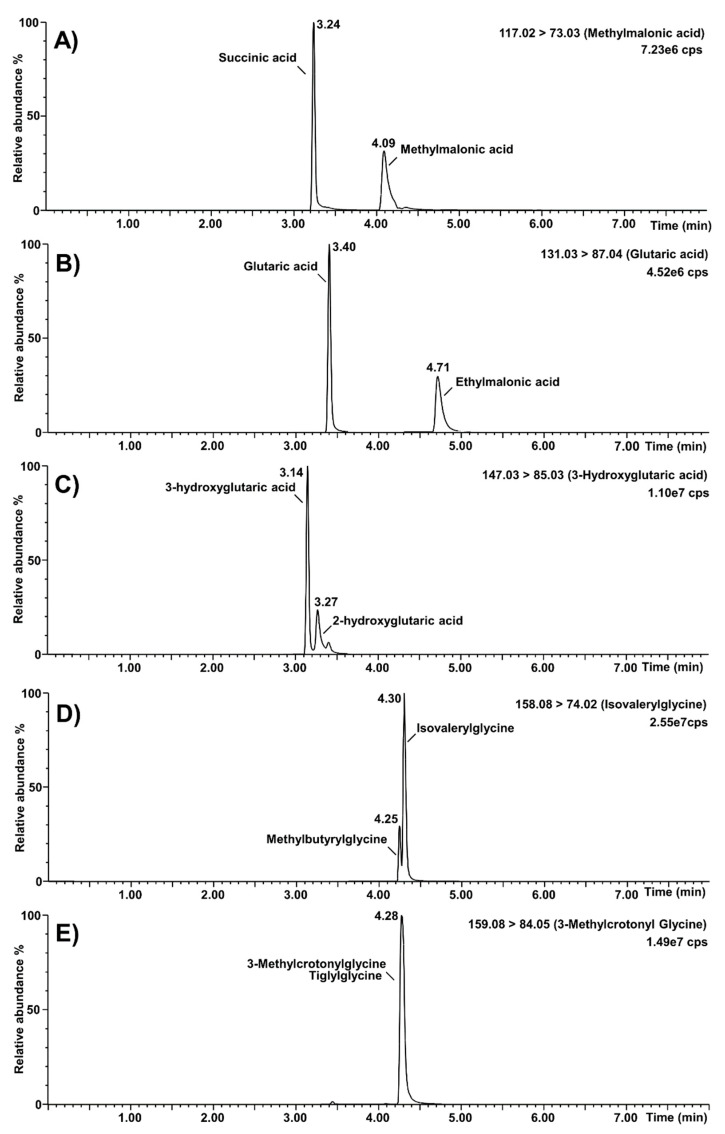
Selectivity of the organic acids second-tier test regarding known isobaric interferences. (**A**): Separation of methylmalonic acid from succinic acid; (**B**): separation of glutaric acid from ethylmalonic acid; (**C**): separation of 3-hydroxyglutaric acid from 2-hydroxyglutaric acid; (**D**): separation of isovalerylglycine from methylbutyrylglycine; (**E**): unseparated peaks of 3-methylcrotonylglycine and tiglylglycine; cps: counts per second.

The specificity of the assay was verified for known isobaric interferences, and results are shown in Figure 5. The high percentage (0.9%) of formic acid in mobile phase A provided a better chromatographic resolution from interferences. Succinic acid (retention time: 3.24 min) was separated chromatographically from methylmalonic acid (retention time: 4.09 min). Similarly, the separation of ethylmalonic acid (retention time: 4.71 min) and glutaric acid (retention time: 3.40 min) was good, as well as for 2-hydroxyglutaric acid (retention time: 3.27 min) and 3-hydroxyglutaric acid (retention time: 3.14 min). Methylbutyrylglycine (retention time: 4.25 min) was almost resolved from isovalerylglycine (retention time: 4.30 min), but not from baseline-to-baseline. Finally, resolution was not achieved for tiglylglycine and 3-methylcrotonylglycine (retention time: 4.28 min). Nevertheless, this will be kept in mind when a positive result is obtained for these biomarkers. It is noteworthy to mention that the MRM function of 3-hydroxypropionic acid was chosen to avoid interference from lactic acid.

### 3.2. Validation

#### 3.2.1. Screening Test

Table S11 summarizes the screening test validation results. For QC1X, QC5X, and the ERNDIM IQA QCs, the intra- and interday RSDs were ≤8.7% except for homogentisic acid, where it was 26.7%. Concerning the intra- and interday accuracy assays for QC1X and QC5X, the bias with the theoretical values were ≤19.3%, except for 3-hydroxypropionic and homogentisic acid, where it was ≤28.0%. It is important to mention that these molecules are the less sensitive of the test, since it was not possible to analyze their most abundant ion due to interferences. Moreover, 3-hydroxypropionic acid is volatile, and homogentisic acid is photosensitive. The intra- and interday accuracy bias for the ERNDIM IQA QCs was ≤12.7%, except for 2-methylcitric acid in the “control organic acid standard”, where it was ≤28.8%. This standard contains an unknown molecule interfering with 2-methylcitric acid, which is not present in urine samples from babies. This interference probably comes from another molecule contained in the standard mixture that is not analyzed by this screening test. Moreover, 3-hydroxyglutaric acid was not analyzed in the ERNDIM IQA QCs due to the interference of the 2-hydroxyglutaric acid also present in high abundance.

#### 3.2.2. Kairos Amino Acid Second-Tier Test

The intraday precision assays were acceptable with %RSDs ranging from 0.3% to 9.4%, and 0.6% to 3.0% for LQC and HQC levels, respectively. Intraday accuracy assays for LQC ranged from −9.3% to 8.3%, except for argininosuccinic acid (bias: 12.3%), carnosine (bias: 21.3%), and anserine (bias: 33.8%). Intraday accuracy assays for the HQC level ranged from −5.6% to 11.4%. Interday precision assays ranged from 0.8% to 9.2%RSD for the LQC (except for argininosuccinic acid at 16.4% and anserine at 18.5%) and from 0.6% to 8.4%RSD for the HQC. The concentrations measured in the control amino acids in serum had biases ranging from −7.8% to 18.9% in level 1, except for cystine (46.0%, 20 vs. 14 µM), and cystathionine (27.3%, 10 vs. 8 µM). In level 2, biases ranged from −4.2 to 14.1, except for cystine (25.0%, 42 vs. 34 µM) and cystathionine (40.2%, 28 vs. 20 µM). These results are shown in Table S12.

Coefficients of determination (*n* = 5 days) were always >0.995, except for anserine (0.993 for day 1). Calibrator 1 had to be removed for methylhistidine, since its concentration was below the LOQ. LODs and LOQs are shown in Table S13.

Matrix effects were assessed, and precision ranged from 1.7% to 7.6%RSD for the LQC, except for glycine (22.9%RSD), probably due to high levels of endogenous glycine. For the HQC, precision ranged from 0.2% to 5.1%RSD. Accuracy was also assessed, and biases ranged from −15.9% to 10.5% in the LQC, except for argininosuccinic acid (bias: 29.2%) and anserine (bias: 52.5%). Biases ranged from −7.8% to 12.2% in the HQC. It is worth mentioning that hydroxyproline, cystathionine, and argininosuccinic acid measures are considered semi-quantitative according to the instructions provided with the kit.

#### 3.2.3. Organic Acids Second-Tier Test

Accuracy and precision assays were measured over three days (*n* = 5 replicates per day) as part of A&P runs. Precision was acceptable with RSDs ranging from 1.0% to 21.0% at LOQ, and from 0.5% to 11.5% for LQC, MQC, and HQC. Accuracy was also acceptable, and biases ranged from −19.6% to 17.9% at LOQ, and from −17.3% to 14.2% for LQC, MQC, and HQC. These results are shown in Table S14.

Other validation runs (*n* = 5) were performed at three levels of concentration (LQC, MQC, HQC) in duplicates. These runs were accepted based on the following criteria recommended by the FDA: ≥67% of QCs with biases at ±15% of their nominal value, and ≥50% of QCs per level with biases at ±15% of their nominal value [25]. Coefficients of determination (*n* = 8 days) were always >0.995. Calibration curves were injected in duplicates, and calibrators with absolute residuals >15% were excluded (20% at LOQ). LODs and LOQs are shown in Table S15. Accuracy and precision assays were acceptable (biases: ≤13.92%; RSDs: ≤10.80%) when a 1:20 dilution factor was performed for specimens with concentrations outside the range of the calibration curve.

Matrix effects were evaluated in six different matrices, precision was acceptable with %RSDs ranging from 3.4% to 9.1%, and accuracy was acceptable with mean biases ranging from −8.6% to 0.0%. Carry-over was verified by the injection of a zero calibrator just after the most concentrated calibrator and did not exceed 20% of LOQ for most compounds, except for malonic acid (54.1% of LOQ), and methylcitric acid (238.3% of LOQ). This carry-over was difficult to eliminate, but nevertheless, precautions were taken to ensure that three blanks were injected after the most concentrated calibrator. The carry-over of methylcitric acid and malonic acid after the injection of the third blank were 47% and 5% of LOQ, respectively. Regarding the selectivity of the assay, there was less than 2% of the IS signals related to interferences present in the urine samples.

Extracted urine specimens collected on filter paper were stable at room temperature (22 °C), and in the refrigerator (4 °C) for at least one week, except for homogentisic acid, which was not stable in the elution solution. In fact, homogentisic acid seems to degrade rapidly when in contact with the NH_4_OH 0.01M elution solution. Nevertheless, considering that high levels of homogentisic acid are found in urine specimens from patients with alkaptonuria, this condition can still be detected by analyzing homogentisic acid as a biomarker. Extracted urine specimens were also stable in a freezer at −30 °C and at −80 °C for at least nine weeks, and after three freeze–thaw cycles. Prepared specimens were stable for at least one week when vials were left in the UHPLC autosampler at 10 °C. The biomarker recoveries from the filter paper ranged from 76% to 98%, depending on the molecule. The recovery of homogentisic acid could not be evaluated considering its high level of degradation.

### 3.3. Reference Values

#### 3.3.1. Screening Test

Figure S1 shows the biomarker levels measured in 8227 random urine samples from the actual NBS program. The guanidineacetic acid/creatine ratio, which is independent of creatinine, was analyzed to better differentiate individuals affected with GAMT.

Table 4 shows a statistical summary of these results, the reference value was established for each biomarker, and the percentage of positive results leading to a second-tier test was determined for each molecule. A RRF of 1.46 was measured between argininosuccinic acid and cystine-D_6_. For uracil, the results presented were from 6697 urine specimens instead of 8227, because its MRM transition was changed during the study due to the interference of an in-source fragment of orotic acid. The cut-off values presented in Table 4 were established using the results obtained from the 8227 urine samples from the NBS Program, the biomarker levels measured in urine specimens from positive cases, and our long-term experience in NBS. Using these cut-off values, the second-tier test occurrences were 1.52% for the Kairos amino acids second-tier test and 6.64% for the organic acids second-tier test.

#### 3.3.2. Kairos Amino Acids Second-Tier Test

Table 5 shows a statistical summary of the biomarker levels measured in 50 urine samples from the urine NBS program. The reference intervals were established from the 5th to 95th percentiles.

#### 3.3.3. Organic Acids Second-Tier Test

Table 6 shows a statistical summary of the biomarker levels measured in 50 urine samples from the urine NBS program. The reference intervals were established from the 5th to 95th percentiles.

### 3.4. Analysis of Positive Cases

**Figure 6:**
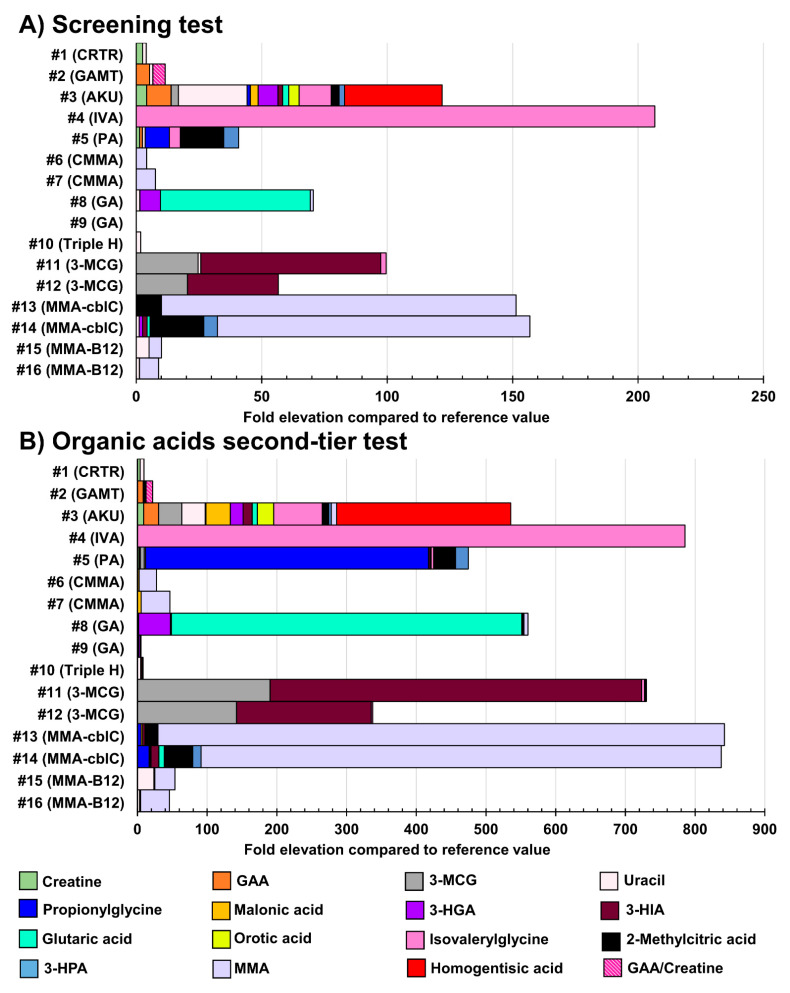
Fold-elevation compared to reference values in urine samples from patients with confirmed organic acidurias, Triple H syndrome, or creatine synthesis and transport disorders (relevant biomarkers targeted by the organic acids second-tier test). Results obtained as part of the screening test are shown in (**A)**, while (**B)** displays results obtained as part of the organic acids second-tier test. The fold-elevation compared to reference values corresponds to the width of the bar for each molecule. CRTR: Creatine transporter deficiency; GAMT: guanidineacetate methyltransferase deficiency; AKU: alkaptonuria; IVA: isovaleric aciduria; PA: propionic aciduria; CMMA: combined malonic and methylmalonic acidemia; GA: glutaric aciduria type 1; 3-MCG: 3-methylcrotonylglycinuria or 3-methylcrotonylglycine; MMA: methylmalonic aciduria or methylmalonic acid; GAA: guanidineacetic acid; 3-HGA: 3-hydroxyglutaric acid; 3-HIA: 3-hydroxyisovaleric acid; 3-HPA: 3-hydroxypropionic acid.

**Figure 7:**
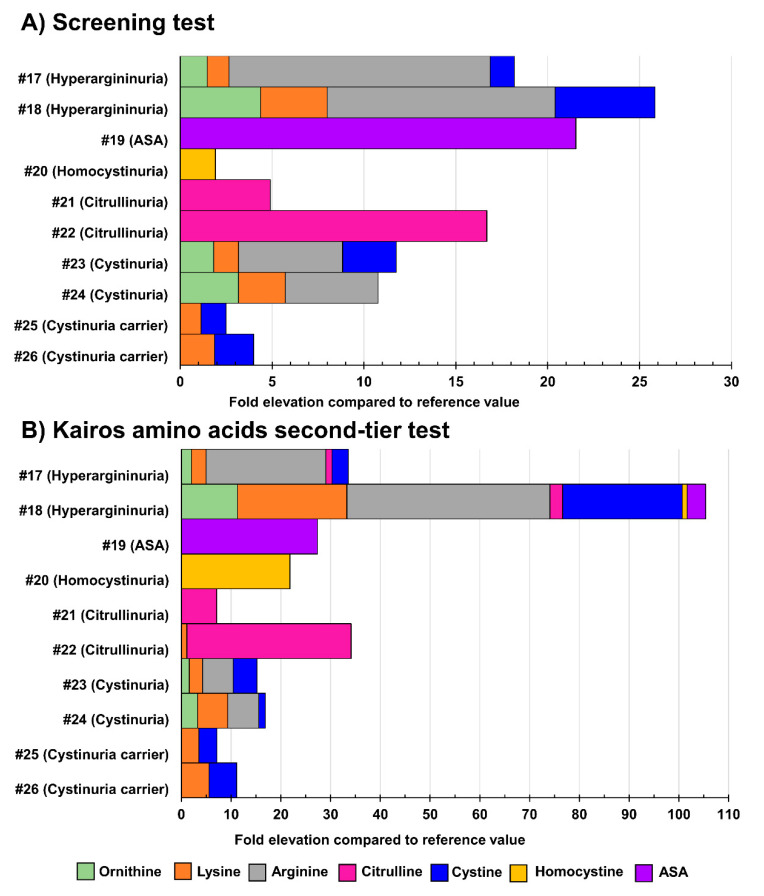
Fold-elevation compared to reference values in urine samples from patients with confirmed aminoacidurias (relevant biomarkers targeted by the Kairos amino acid second-tier test). Results obtained as part of the screening test are shown in (**A**), while (**B**) displays results obtained as part of the Kairos amino acid second-tier test. The fold-elevation compared to reference values corresponds to the width of the bar for each molecule. ASA: argininosuccinic aciduria.

Table S16 displays the biomarker levels measured in urine specimens from positive cases suffering from the IEM targeted by the NBS. Unfortunately, due to their rarity, urine samples from newborn babies were not available for all the IEM under study. For those unavailable IEM in newborns, urine samples from older children and adults were analyzed. Figure 6 shows the results obtained in urine samples from patients with confirmed organic acidurias, Triple H syndrome, or creatine synthesis and transport disorders, while Figure 7 shows results obtained in urine samples from patients having confirmed aminoacidopathies.

**Figure 8:**
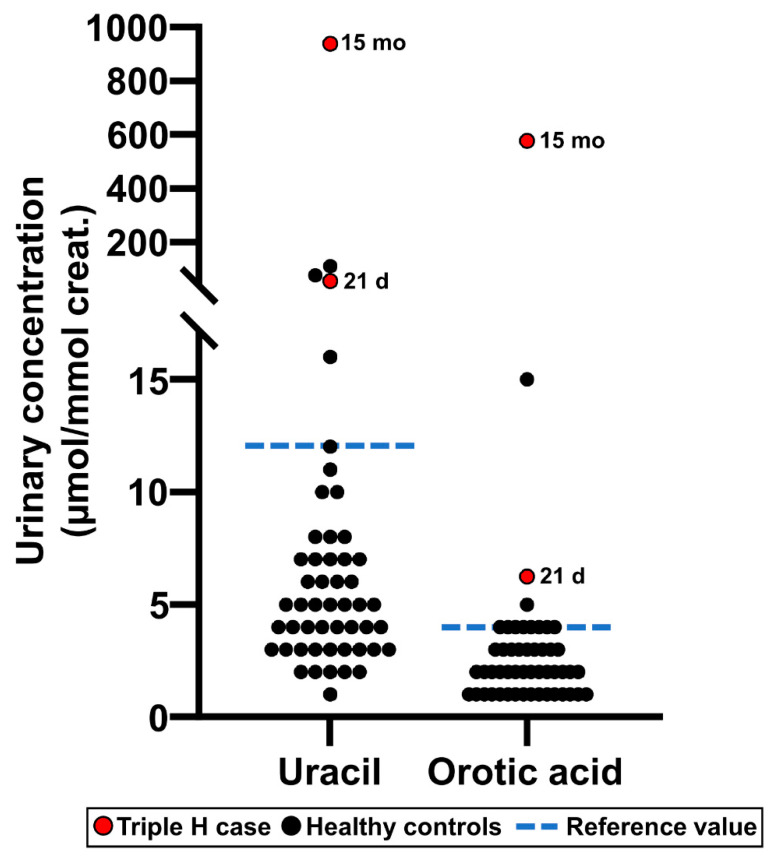
Uracil and orotic acid urinary concentrations measured as part of the Organic acids second-tier test in a patient affected with Triple H syndrome, both at 21 days of age (this specimen was collected as part of the newborn screening program) and at 15 months of age. Values measured in healthy reference controls (*n* = 50) are also shown for comparison purposes.

The results following the analysis of urine specimens obtained from a patient affected with Triple H syndrome are shown in Figure 8. The patient was diagnosed at 15 months of age, and the treating geneticist asked us to retrieve the filter paper from the NBS program which was initially collected at 21 days of age and stored at −20 °C. All filter papers are stored for a duration of five years. It is noteworthy to mention that Triple H syndrome is not screened as part of the current program performed on TLC.

### 3.5. Comparison of Methods for Amino Acids and Organic Acids

A comparison of the different methodologies for amino acids and organic acids was performed to evaluate the results obtained for urine filter paper samples showing borderline results by thin layer chromatography at the screening program. Table S17 shows the results for some of the amino acids (number of samples: 87) analyzed by: (1) the current MS/MS screening method; (2) the Biochrom 30 Amino Acid Analyzer related methodology using ion-exchange chromatography; and (3) the second-tier MS/MS test with the Kairos system. For the organic acids (number of samples: 210) (Table S18), the comparison of the methodologies was done for urine filter paper samples with: (1) the current MS/MS screening method; and (2) the GC/MS methodology performed in the biochemical genetics laboratory using the BSTFA derivatization reaction [27]; (3) the second-tier MS/MS test. The results were normalized to the creatinine value, which was done according to three different methods: MS/MS for the screening test, the Jaffe colorimetric method [28], and the current second-tier test (MS/MS) for organic acids.

## 4. Discussion

Over the years, different studies revealed that using a urine matrix for screening IEM is efficient for early detection of cases, some of which might not have been detected in DBS at one or two days of age. In fact, Pitt et al. reported that <2% of urine specimens received in his laboratory, and typically in other biochemical genetics laboratories, tested positive for an IEM [29]. NMR spectroscopy has also been used for the detection of 75 IEM in urine specimens from newborns [30]. A metabolomic study revealed the feasibility to detect efficiently urine samples of diagnosed patients with IEM (*n* = 34) compared to controls (*n* = 66) [31]. The authors also evaluated two normalization variables, creatinine and osmolality, for each biomarker analyzed. The results using the two approaches to normalization of biomarkers were comparable. Moreover, they reported no effect of the normalization calculation on the identification of biomarkers or group of biomarkers [31]. Another screening program reported that urine contributed significantly to detect IEM in 44% of the cases investigated [32]. These authors mentioned that both blood and urine were collected from 99.9% of newborns on the third day of life as part of an expanded screening program in the Galacia region of Spain. They also emphasized that urine specimens allowed the development of second-tier tests, which included more specific biomarkers than those observed in blood. Moreover, some IEM such as Triple H syndrome, cystinuria, and alkaptonuria are not expressed in blood but have high levels of biomarker excretion in urine.

In our case, the TLC technique used for more than 48 years has been reliable for the qualitative detection of urinary aminoacidopathies, as well as organic acidurias at a low cost (~5.00$/newborn). However, the advancement of mass spectrometry technologies now offers the possibility to target additional biomarkers, such as orotic acid and uracil for Triple H syndrome, which cannot be detected by the TLC technique and proven not to be detectable in DBS from newborns [9], and it also allows the absolute quantitation of biomarkers normalized to creatinine.

The results from the research project undertaken to evaluate the feasibility of a technological transfer from the TLC method to mass spectrometry for the analysis of more than 70,000 urine specimens per year have proven positive. We have developed and validated a rapid 2.85 min flow injection mass spectrometry method for the quantitative analysis of 22 biomarkers and appropriate reference standards, comprising aminoacidurias, organic acidurias, and creatine transport and synthesis disorders, normalized to creatinine in a single multiplex analysis. Five hundred urine filter paper samples can be analyzed per run in less than 24 h, with high and low-quality controls, as well as positive control cases for each batch.

In addition, second-tier test methodologies for amino acids and other biomarkers, including selected organic acids, have been developed and validated to reduce the number of requests for a repeat urine specimen from the parents. These latter methods are rapid, reliable, efficient, and robust. They can also be applicable in biochemical genetics laboratories worldwide either for mass or high-risk screening.

The comparison of the different methodologies using also different creatinine methods was found to be difficult to interpret because of the variability in the creatinine values obtained with the three methods. Nevertheless, the interpretation of the results was similar in most of the samples analyzed by the quantitative methods.

As shown over the years, the use of urine samples collected on filter paper represents an easier way to collect biological specimens for NBS and favors the storage and shipment of samples at lower cost without degradation of the biomarkers targeted.

In conclusion, the infrastructure and the methodologies are already in place waiting for the final approval of the Ministry of Health and Social Services decision for the technological transfer from TLC to mass spectrometry. Considering the number of disorders that could be detected by this novel mass spectrometry urine screening method compared to the cost of having a child severely impaired, if not detected, would be important assets to consider.

## Figures and Tables

**Table 1:** Targeted inborn errors of metabolism and associated biomarkers.

**Disorders**	**OMIM No.**	**Targeted Biomarkers**
**Organic acidurias**		
Methylmalonic aciduria(s)		
*cblA type*	251,100	Methylmalonic acid↑, 2-Methylcitric acid↑
*cblB type*	251,110	Methylmalonic acid↑, 2-Methylcitric acid↑
*cblC type*	277,400	Methylmalonic acid↑, 2-Methylcitric acid↑, Homocystine↑
*cblD type*	277,410	Methylmalonic acid↑, 2-Methylcitric acid↑, Homocystine↑
*cblF type*	277,380	Methylmalonic acid↑, 2-Methylcitric acid↑, Homocystine↑
*mut type*	251,000	Methylmalonic acid↑, 2-Methylcitric acid↑
Combined malonic and methylmalonic aciduria (CMAMMA)	614,265	Methylmalonic acid↑, 2-Methylcitric acid↑, Malonic acid↑
Propionic aciduria	606,054	Propionylglycine↑, 2-Methylcitric acid↑, 3-Hydroxypropionic acid↑
Isovaleric aciduria	243,500	Isovalerylglycine↑, 3-Hydroxyisovaleric acid↑
Glutaric aciduria type 1	231,670	Glutaric acid↑, 3-Hydroxyglutaric acid↑
Alkaptonuria	203,500	Homogentisic acid↑
3-Methylcrotonylglycinuria type I	210,200	3-Methylcrotonylglycine↑, 3-Hydroxyisovaleric acid↑
**Urea cycle disorders**		
Argininosuccinic aciduria	207,900	Argininosuccinic acid↑
Hyperargininuria	207,800	Arginine↑
Citrullinuria, classic	215,700	Citrulline↑
Citrullinuria type II	605,814	Citrulline↑
Triple H syndrome	238,970	Orotic acid↑, Uracil↑,
**Disorders of amino acid transport**		
Cystinuria	220,100	Cystine↑, Ornithine↑, Lysine↑, Arginine↑
**Disorders of amino acid metabolism**		
Homocystinuria	236,200	Homocystine↑
**Creatine synthesis and transport disorders**		
Guanidinoacetate methyltransferase deficiency (GAMT)	601,240	Creatine↓, Guanidineacetic acid↑
Arginine:glycine amidinotransferase deficiency (AGAT)	602,360	Creatine↓, Guanidineacetic acid↓
Creatine transporter deficiency (CRTR)	300,352	Creatine↑

↑: elevation of biomarker; ↓: diminution of biomarker.

**Table 2:** UHPLC and MS parameters for the screening test.

**UHPLC Parameters**	
Analysis mode	Flow injection (no chromatography)
Mobile phase	95:5 H_2_O:ACN + 0.1% F.A. (isocratic)
Weak wash solvent	H_2_O + 0.1% F.A.
Strong wash solvent	H_2_O + 0.1% F.A.
Injection volume	10 µL
Injector type	Flow through needle
Flow rates	0.00 → 0.22 min: 0.100 mL/min
	0.22 → 1.20 min: 0.015 mL/min
	1.20 → 2.00 min: 0.500 mL/min
**MS Parameters**	
Ionization mode	Electrospray (ESI)
Acquisition mode	Multiple reaction monitoring (MRM)
Capillary Voltage	ESI(−): 2.00 kV; ESI(+): 2.00 kV
Desolvation temperature	200 °C
Desolvation gas flow	1000 L/h
Cone gas flow	10 L/h
Source temperature	150 °C
Span	0.1 Da
Dwell time	14 ms

**Table 3:** UHPLC and MS parameters for the Organic Acids second-tier test.

**UHPLC Parameters**	
Column	Atlantis PREMIER BEH C_18_ AX VanGuard FIT
ID x Length	2.1 × 100 mm
Particle size	1.7 µm
Column temperature	30 °C
Mobile phase A	40:60 ACN:H_2_O 30 mM Amm. Form. + 0.9% F.A.
Mobile phase B	95:5 H_2_O:ACN
Gradient	
*Flow rate (mL/min)*	*Mobile phase %*
0.350	0.00 → 1.50 min: 100%B
0.350	1.50 → 5.00 min: 100 → 0%B (linear)
0.350	5.00 → 7.00 min: 0%B
0.350	7.00 → 7.10 min: 0 → 100%B (linear)
0.350	7.10 → 8.00 min: 100%B
Weak wash solvent	H_2_O
Strong wash solvent	H_2_O
Injection volume	10 µL
Injector type	Flow through needle
Autosampler temperature	10 °C
**MS Parameters**	
Ionization mode	Electrospray (ESI)
Acquisition mode	Multiple reaction monitoring (MRM)
Capillary Voltage	ESI(+): 0.50 kV; ESI(−): 0.60 kV
Desolvation temperature	500 °C
Desolvation gas flow	750 L/h
Cone gas flow	0 L/h
Source temperature	150 °C
Span	0.1 Da

**Table 4:** Statistical summary of the screening test performed on urine samples from the Newborn Screening Program (*n*= 8227), normal cut-off values for the different biomarkers, and their positive rate. For uracil *n*= 6697. Creatinine ratio = ratio of the creatinine signal obtained with fragments at *m/z* 44 and 86. Creat. = creatinine.

Biomarker	Median	1st Centile	99th Centile	99.9th Centile	Cut-Off Min.	Cut-Off Max.	Positive Rate
	µmol/mmol Creat.	µM	µmol/mmol Creat.	µmol/mmol Creat.	µM	µmol/mmol Creat.	%
Creatinine (µM)	78.88	22.91	--	--	0.6	--	0.08
Creatinine ratio (no unit)	1.28	1.19	1.39	1.42	1.15	1.51	0.46
Creatine	185.68	--	800.49	1057.59	--	1000	0.18
Guanidineacetic acid	127.03	--	249.89	319.71	--	290	0.30
Guanidineacetic acid/creatine ratio (no unit)	0.72	--	3.53	4.47	--	4.40	0.13
3-Methylcrotonylglycine	7.68	--	29.98	70.37	--	60	0.18
Uracil	26.83	--	78.36	150.68	--	55	4.87
Propionylglycine	2.58	--	13.20	31.00	--	40	0.09
Malonic acid	32.81	--	114.92	364.74	--	200	0.22
3-Hydroxyglutaric acid	20.34	--	39.24	61.61	--	60	0.15
3-Hydroxyisovaleric acid	14.58	--	59.50	113.48	--	150	0.05
Glutaric acid	23.17	--	58.38	109.90	--	200	0.05
Orotic acid	11.20	--	35.00	97.63	--	43	0.63
N-Isovalerylglycine	1.52	--	4.31	6.87	--	15	0.05
2-Methylcitric acid	6.56	--	16.74	32.22	--	38	0.09
3-Hydroxypropionic acid	13.38	--	89.30	259.18	--	140	0.45
Methylmalonic acid	8.37	--	104.29	250.78	--	150	0.55
Homogentisic acid	2.87	--	11.14	16.90	--	100	0.00
Ornithine	43.42	--	267.96	564.87	--	350	0.49
Lysine	289.74	--	873.09	1441.75	--	1100	0.40
Arginine	25.71	--	131.89	254.65	--	200	0.33
Argininosuccinic acid	4.17	--	11.82	18.54	--	50	0.00
Citrulline	21.28	--	172.08	391.92	--	250	0.42
Cystine	27.15	--	138.36	339.19	--	200	0.43
Homocystine	2.02	--	6.00	13.28	--	14	0.12

**Table 5:** Reference intervals (5th–95th percentiles) established for each biomarker analyzed as part of the Kairos amino acids second-tier test, following the analysis of 50 urine samples from 21-day old babies. Minimum, maximum, and median values are also displayed.

Biomarker	Minimum	Maximum	Median	5th Percentile	95th Percentile
	µmol/mmol Creat.	µmol/mmol Creat.	µmol/mmol Creat.	µmol/mmol Creat.	µmol/mmol Creat.
Taurine	58	1362	645	181	1253
Aspartic acid	0	880	51	9	235
Hydroxyproline	65	1173	420	139	949
Threonine	28	741	160	54	549
Serine	0	1520	451	238	1145
Asparagine	0	237	67	0	170
Glutamic acid	11	275	46	15	193
Glutamine	103	691	301	144	533
Sarcosine	0	29	0	0	15
Alpha Aminoadipic Acid	0	104	13	0	57
Proline	47	773	210	56	643
Glycine	435	3366	1762	759	2781
Alanine	139	837	343	154	733
Citrulline	5	126	29	9	106
Alpha Aminobutyric Acid	0	0	0	0	0
Valine	3	220	33	12	137
Cystine	8	99	30	11	80
Methionine	0	52	0	0	15
Homocitrulline	0	93	0	0	52
Allo-Isoleucine	0	0	0	0	0
Cystathionine	0	21	0	0	10
Isoleucine	0	1075	10	0	66
Leucine	0	274	29	4	103
Argininosuccinic Acid	0	80	9	0	42
Tyrosine	0	150	58	24	104
Beta Alanine	0	105	27	3	85
Phenylalanine	0	103	21	3	53
Beta Aminoisobutyric Acid	0	943	8	0	226
Homocystine	0	0	0	0	0
Gamma Aminobutyric Acid	6	54	19	7	44
L-Ornithine	2	376	42	9	257
Lysine	11	505	121	37	282
1-Methyl Histidine	0	156	0	0	96
Histidine	0	371	177	0	319
3-Methyl Histidine	0	133	0	0	50
Carnosine	0	222	74	0	157
Arginine	25	210	49	27	110
Ethanolamine	55	983	208	89	566
Phosphoethanolamine	0	60	13	0	45
Hydroxylysine	0	40	10	0	26
Glycyl proline	0	51	19	0	36
S-Sulfocysteine	0	0	0	0	0
Anserine	0	80	30	0	66
Kynurenine	0	0	0	0	0
Tryptophan	10	66	30	15	53

**Table 6:** Reference intervals (5th–95th percentiles) established for each biomarker analyzed as part of the organic acids second-tier test, following the analysis of 50 urine samples from 21-day old babies. Extreme outliers (>3 interquartile ranges from the 75th percentile) were excluded from the statistics. Minimum, maximum, and median values are also displayed.

Biomarker	Minimum	Maximum	Median	5th Percentile	95th Percentile
	µmol/mmol Creat.	µmol/mmol Creat.	µmol/mmol Creat.	µmol/mmol Creat.	µmol/mmol Creat.
Guanidineacetic acid	56	257	108	64	187
Guanidineacetic acid/creatine ratio (no unit)	0.23	3.74	0.88	0.25	3.18
3-Methylcrotonylglycine	1	9	3	1	8
Creatine	23	755	144	26	581
Creatinine	44	270	127	51	228
Orotic acid	1	5	2	1	4
Methylmalonic acid	0	33	6	0	26
Glutaric acid	0	41	8	0	26
3-Hydroxyisovaleric acid	1	52	10	2	29
3-Hydroxyglutaric acid	0	12	2	0	9
Malonic acid	1	29	6	2	23
Isovalerylglycine	0	4	2	0	4
2-Methylcitric acid	0	29	8	0	23
Uracil	1	16	5	2	12
Propionylglycine	0	1	0	0	1
3-Hydroxypropionic acid	6	68	17	7	62
Homogentisic acid	0	2	0	0	2

## Supplementary Materials

The following are available online at https://www.mdpi.com/2409-515X/7/1/18/s1, Figure S1: Biomarker levels obtained from 8277 control urine specimens from newborn babies during screening analysis, Table S1: Standard stock solution concentrations, Table S2: Kairos Amino Acid kit working solution concentrations, Table S3: Working solution concentrations for the Organic acids second-tier test, Table S4: Preparation of the internal standard (IS) mixture for the screening test., Table S5: Preparation of the internal standard working solution for the Organic acids second-tier test, Table S6: Metabolite concentrations contained in the in-house quality controls (QC) corresponding approximately to one time (QC1X) and five times (QC5X) the normal reference values, Table S7: Mass spectrometry transitions for the analysis of the biomarkers in the Multiple Reaction Monitoring (MRM) mode for the first tier test, Table S8: UHPLC and MS parameters for the Kairos amino acid second-tier test, Table S9: Mass spectrometry functions for the analysis of the biomarkers in the Multiple Reaction Monitoring (MRM) mode (positive electrospray) for the Kairos amino acid second-tier test. Internal standards (IS) are in bold, Table S10: Mass spectrometry functions for the analysis of the biomarkers in the Multiple Reaction Monitoring (MRM) mode (positive and negative electrospray) for the Organic acids second-tier test, Table S11: Intra- and interday precision assays (*n* = 5) and accuracy assays (*n* = 5) for the screening test measured in quality controls, Table S12: Intraday (*n* = 5 replicates) and interday (*n* = 5 days) precision assays and intraday accuracy assays (*n* = 5 replicates) measured as part of the validation of the Kairos amino acid second-tier test, Table S13: Limits of detection (LOD) and limits of quantitation (LOQ) for the Kairos test, Table S14: Precision and accuracy of the Organic acids second-tier test, as measured during A&P runs, Table S15: Limits of detection (LOD) and limits of quantitation (LOQ) for the Organic acids second-tier test, Table S16: Biomarker levels in urine samples from positive cases of inborn errors of metabolism targeted by the newborn screening program, Table S17: Analysis of amino acids in urine filter paper samples (*n* = 87) showing borderline results by thin layer chromatography at the screening program using three different methods, Table S18: Analysis of organic acids in urine filter paper samples (*n* = 210) showing borderline results by thin layer chromatography at the screening program using three different methods.

## Abbreviations

NBS	Newborn screening
IEM	Inborn errors of metabolism
QNGM	Quebec Network of Genetic Medicine
DBS	Dried blood spot
DUS	Dried urine spot
TLC	Thin layer chromatography
Triple H	Hyperornithinemia-hyperammonemia-homocitrullinuria
QNSP	Quebec Neuroblastoma Screening Project
NIH	National Institutes of Health
HVA	Homovanillic acid
VMA	Vanillylmandelic acid
QCs	Quality controls
MS/MS	Tandem mass spectrometry
LC-MS/MS	Liquid chromatography-tandem mass spectrometry
CID	Collision-induced dissociation
IS	Internal standard
REB	Research Ethics Board
CIUSSSE-CHUS	Centre intégré universitaire de santé et de services sociaux de l’Estrie-Centre hospitalier universitaire de Sherbrooke
CMMA	Combined malonic and methylmalonic aciduria
ASA	Argininosuccinic aciduria
HCl	Hydrochloric acid
IQA	Internal quality assurance
ERNDIM	European Research Network for Evaluation and Improvement of Screening, Diagnosis, and Treatment of Inborn Errors of Metabolism
LOQ	Limit of quantitation
UHPLC-MS/MS	Ultra-high performance liquid chromatography tandem mass spectrometry
MRM	Multiple reaction monitoring
RRF	Relative Response Factor
LOD	Limits of detection
BSTFA	N,O-Bis(trimethylsilyl)trifluoroacetamide
