# Supplementary material for: Neonatal Urine Screening Program in the Province of Quebec: Technological Upgrade from Thin Layer Chromatography to Tandem Mass Spectrometry
Source: Int J Neonatal Screen. 2021 Mar 20;7(1):18. doi: 10.3390/ijns7010018 (PMC8006232; doi:10.3390/ijns7010018)
Supplement: Supplementary file 1 [file IJNS-07-00018-s001.zip › Figure S1.pptx]

## Slide 1
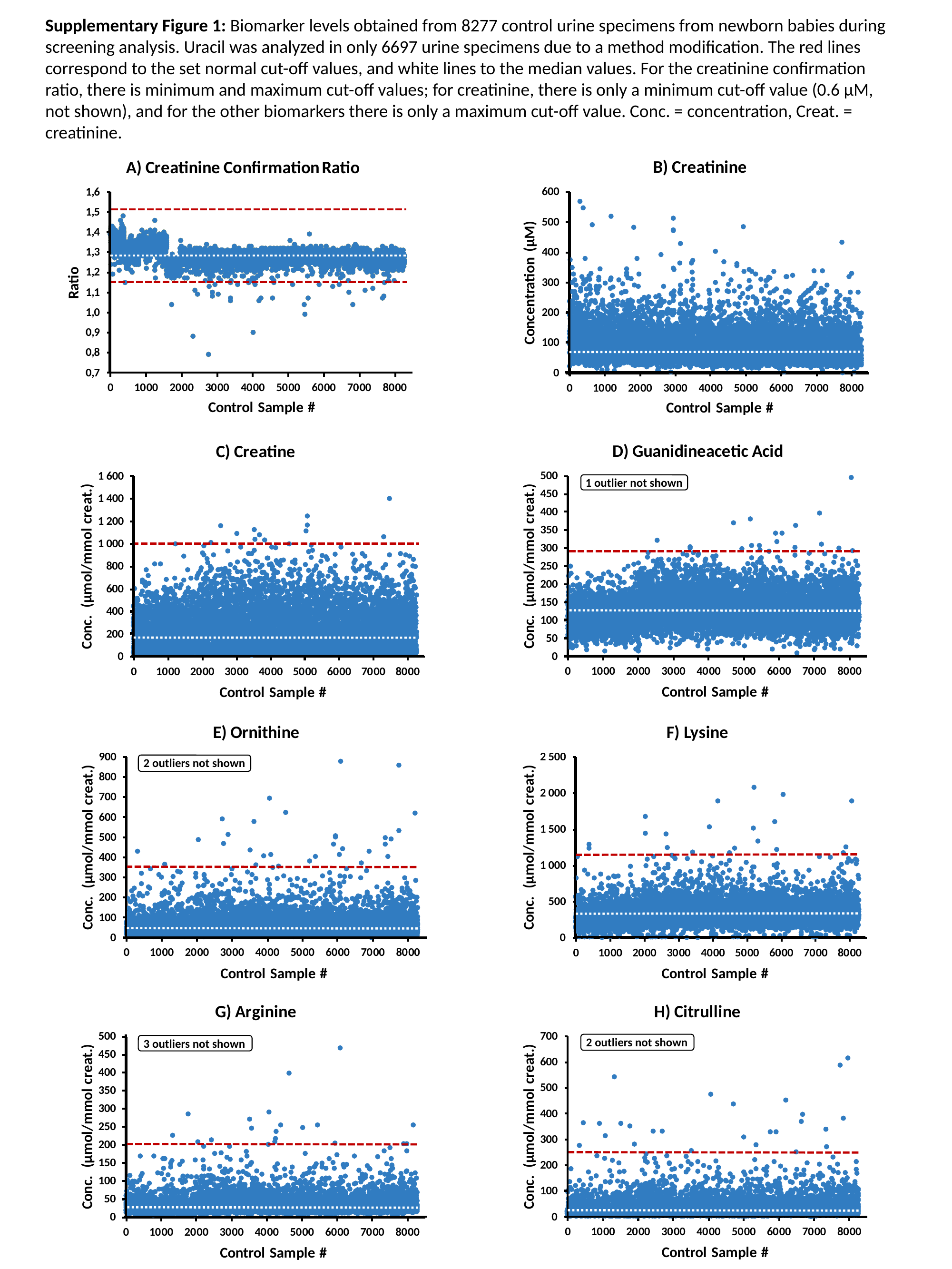

Supplementary Figure 1: Biomarker levels obtained from 8277 control urine specimens from newborn babies during screening analysis. Uracil was analyzed in only 6697 urine specimens due to a method modification. The red lines correspond to the set normal cut-off values, and white lines to the median values. For the creatinine confirmation ratio, there is minimum and maximum cut-off values; for creatinine, there is only a minimum cut-off value (0.6 µM, not shown), and for the other biomarkers there is only a maximum cut-off value. Conc. = concentration, Creat. = creatinine.
1 outlier not shown
2 outliers not shown
2 outliers not shown
3 outliers not shown

## Slide 2
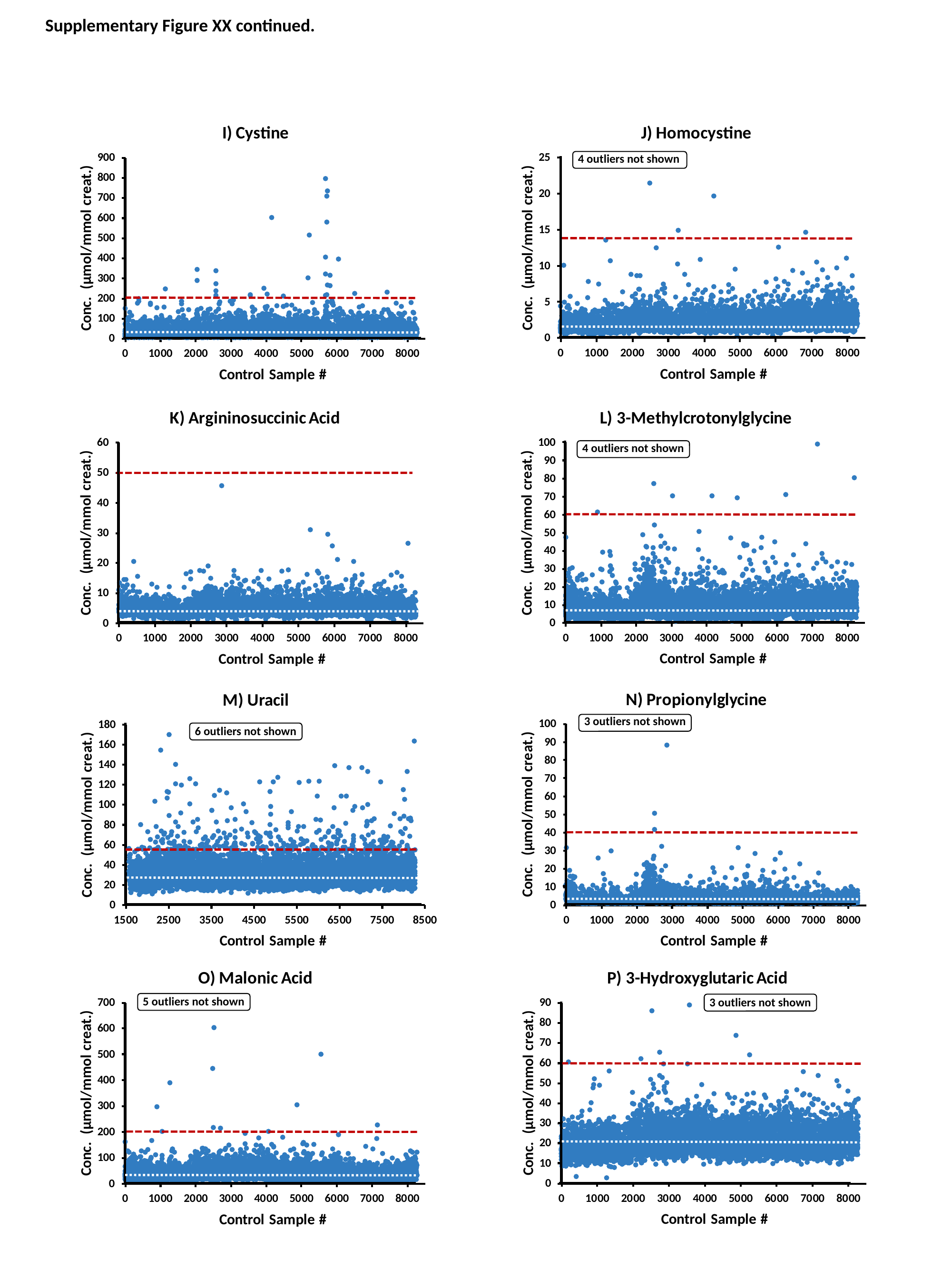

Supplementary Figure XX continued.
4 outliers not shown
4 outliers not shown
6 outliers not shown
3 outliers not shown
3 outliers not shown
5 outliers not shown

## Slide 3
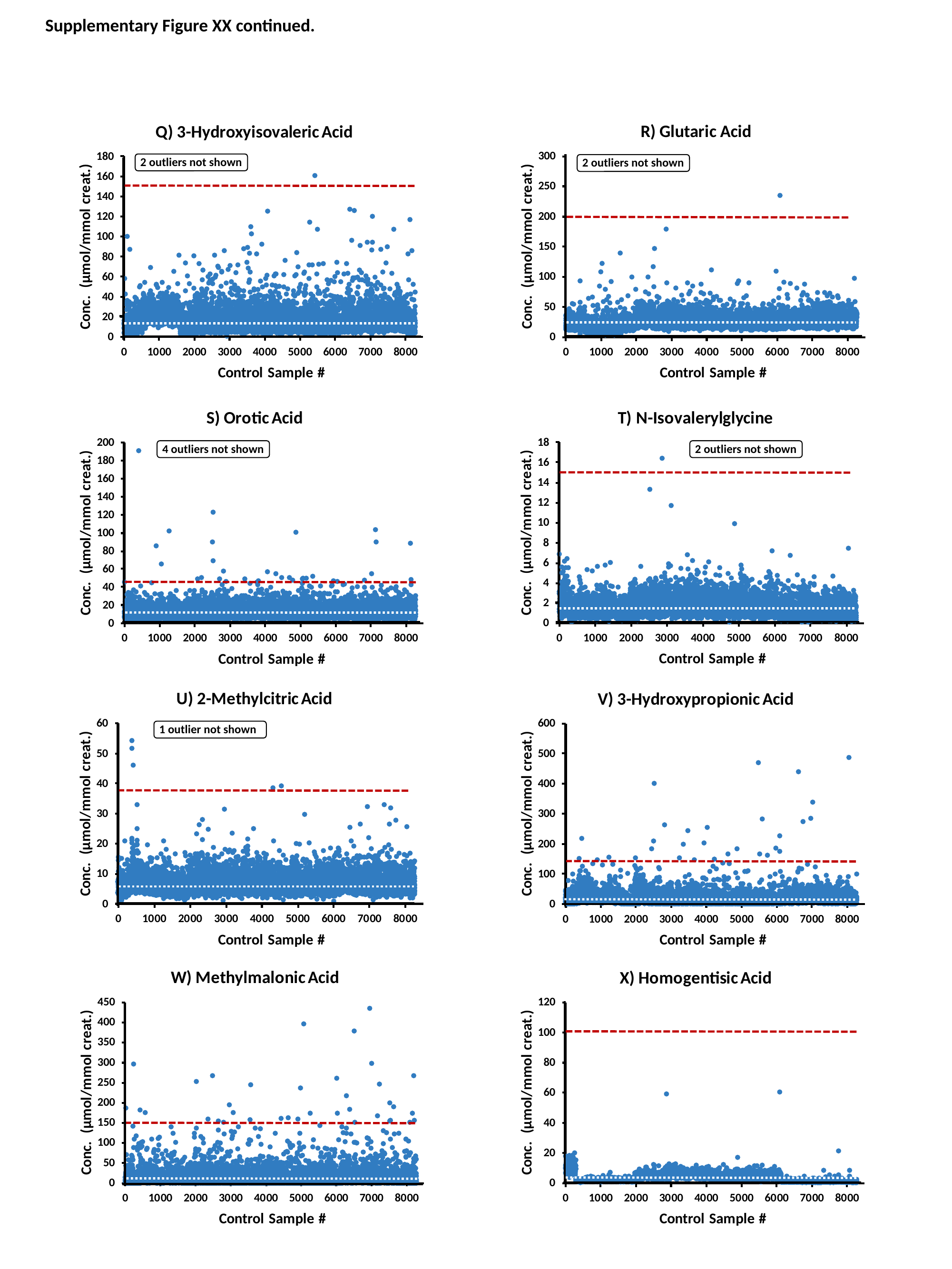

Supplementary Figure XX continued.
2 outliers not shown
2 outliers not shown
2 outliers not shown
4 outliers not shown
1 outlier not shown

## Slide 4
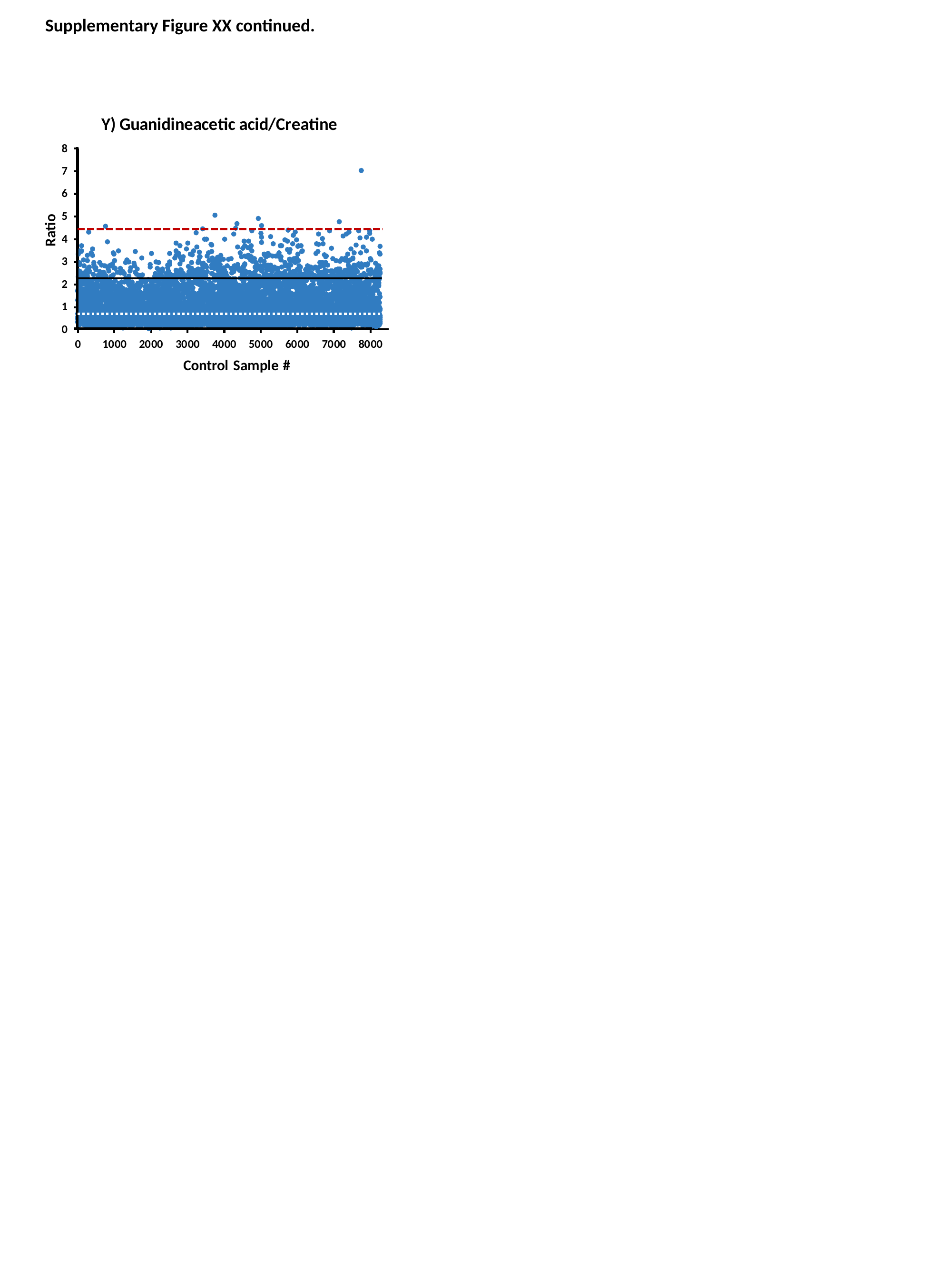

Supplementary Figure XX continued.
